# Determination of low environmental free cyanide concentrations in freshwaters

**DOI:** 10.1007/s11356-020-12062-7

**Published:** 2020-12-17

**Authors:** Burkhard Knopf, Heinz Rüdel, Dirk Hansknecht, Thorsten Klawonn, Knut Kreuzer

**Affiliations:** 1 Fraunhofer Institute for Molecular Biology and Applied Ecology IME, Schmallenberg, Germany; 2 Röhm GmbH, Deutsche-Telekom-Allee, Darmstadt, Germany

**Keywords:** Free cyanide, Continuous flow analysis, Environmental quality standard, Surface water, Environmental monitoring, Compliance monitoring, Water framework directive

## Abstract

Cyanide compounds are naturally emitted into the environment in low levels by degradation processes or emitted from anthropogenic sources. In surface water, complex cyanide compounds as well as “free cyanide” are present. The latter term covers hydrogen cyanide and cyanide compounds which easily liberate hydrogen cyanide under slightly acidic conditions. Especially free cyanide may cause adverse effects in the environment. To exclude negative impacts on freshwater systems, in the context of the European Water Framework Directive (WFD), preventive regulatory activities for free cyanide are currently under discussion. However, established analytical methods for quantification of free cyanide only obtain limits of quantification (LOQs) in the range of 1 μg L^−1^. Thus, these methods are not sufficiently sensitive for a potential environmental quality standard (EQS) compliance monitoring at water concentrations below the current predicted no effect concentration (PNEC) level of free cyanide. In the present study, a standardized continuous flow analysis (CFA) method for quantification of low free cyanide concentrations was adapted by applying a special system which allows an ultra-sensitive photometric detection of a colored cyanide derivative. By this means, LOQs in a range of one magnitude below the PNEC are achievable. The method was validated according to ISO/IEC 17025 requirements. Free cyanide concentrations in tested surface water samples from a small river and a barrier lake with low anthropogenic influences were very low and clearly below the PNEC. The results prove that the adapted CFA method is suitable for the analysis of low concentration free cyanide in freshwaters and appropriate for a possible EQS compliance monitoring.

**Supplementary Information:**

The online version contains supplementary material available at 10.1007/s11356-020-12062-7.

## Introduction

In environmental waters, cyanide can occur as entity in simple and complex compounds (Jaszczak et al. 2017). Free cyanide, also designated as easily liberatable cyanide, is defined as the sum of cyanide ions and cyanide bound in weak metal cyanide complexes that liberate hydrogen cyanide at a pH of about 4 (ISO 2012). It exists in fresh water systems in low concentrations and originates from naturally occurring degradation processes of plants and microbes. In contrast to information on total cyanide covering simple and complex compounds, information on background levels of the toxicological relevant free cyanide in freshwater systems is extremely sparse. Krutz (1981), for example, analyzed free cyanide in the range of 0.1–10 μg L^−1^ in two small rivers with seasonal fluctuations in Germany. Beside natural origins, cyanide compounds may be emitted from anthropogenic sources, like mining operations, metallurgic industry, and wastewater treatment plants or during fires (Jaszczak et al. 2017).

Free cyanide anions are highly toxic towards aquatic organisms ((Australian Water Association (2000); (ECETOC 2007)). For example, the current predicted no effect concentration (PNEC) level for freshwater organisms under European chemicals’ management directive REACh is as low as 1 μg L^−1^ (ECHA 2020). The European Water Framework Directive 2000/60/EC (WFD) established a “targeted EU-wide monitoring of substances of possible concern” for the aquatic environment via a watch list mechanism (EU 2013; EU 2015a). Consultations on such a multi-annual monitoring for free cyanide are currently ongoing and, below other aspects, refer to the current unavailability of a reliable analytical method of sufficient sensitivity, i.e., one order of magnitude below the PNEC (EU 2015b; Loos et al. 2018; Peters et al. 2012). This concentration range is considered relevant for a potential future environmental quality standard (EQS) in several evaluations (Sorokin et al. 2012) (EU 2015b; Peters et al. 2012) which should prevent adverse effects by androgenic released substances towards pelagic communities of freshwater organisms. The respective WFD compliance monitoring requires analytical methods with a limit of quantification (LOQ) of at least 30% of the EQS and a maximum measurement uncertainty of 50% to ensure the reliable quantification of target compounds in waters (EC 2009). Thus, there is a strong demand for an analytical method allowing free cyanide determinations in this low concentration range.

Since present analytical methods for free cyanides (e.g., spectrometric and electrochemical methods as well as mass spectrometry, ion and gas chromatographic procedures) only can achieve LOQs above 1 μg L^−1^ (see e.g., review by Ma and Dasgupta (2010)), a more sensitive procedure for the determination of low levels of free cyanide was required. To this end, a standardized continuous flow analysis (CFA) method was adapted to a higher sensitivity, validated in the laboratory and with samples from a river and a barrier lake, and implemented for routine application. The optimized CFA method with photometric quantification is based on international standard ISO 14403-2 (ISO 2012) and has an application range of 10–100 μg L^−1^.

The validation and application of the adapted CFA method for the quantification of low levels of free cyanide in surface waters according to WFD technical requirements were initiated and funded by a consortium of European industry associations (CONCAWE, Cefic, Eurofer, and Euromines).

## Materials and methods

### Instrumentation

As device for the sensitive determination of free cyanide, the CFA system San++ Automated Wet Chemistry Analyzer (Skalar Analytical B.V., Breda, The Netherlands), including an autosampler and a computer with a dedicated software for the calculation of free cyanide concentrations of samples, was chosen. This instrument allows an automatic distilling of free cyanide from aqueous samples for separation and enrichment of the cyanide prior to a chemical reaction forming a dye for the subsequent photometric detection. A special cuvette (glass fiber) with a length of 50 cm was installed to improve the sensitivity: According to the Beer-Lambert law, the layer thickness is proportional to the extinction (absorbance of the dye) thus improving the sensitivity by a factor 50 in comparison to a CFA instrument with a standard 1-cm cuvette. The standard ISO 14403-2 (ISO 2012) explicitly mentions the option to enhance the sensitivity of the method by applying a detection cell with a larger optical path length so that the instrument is compliant with the standard.

### Standards and reagents

Principally, for CFA, the use of a tenside in the carrier solution is required for separating discrete volumes by air segments (bubbles). However, based on pre-tests, the non-ionic tenside polyoxyethylene dodecylether (CAS No. 9002-92-0) originally proposed in standard ISO 14403-2 (ISO 2012) was substituted by the ionic tenside dodecyl diphenyloxide disulphanated sodium salt (CAS No. 119345-04-9; trade name FFD6). FFD6 was more suitable for maintaining a low and stable background of the applied CFA instrument with the 50-cm cuvette than polyoxyethylene dodecylether. FFD6 seems generally appropriate as tenside for this purpose and already is used in another CFA application (for ortho-phosphate analysis, ISO 15681-2).

Reagents were of “for analysis” quality: citric acid monohydrate (purchased from Honeywell Fluka, via Fisher Scientific GmbH, Schwerte, Germany), zinc sulfate heptahydrate (Merck KGaA, Darmstadt, Germany), potassium hydrogen phthalate (Merck), FFD6 (provided as 25–50% solution in water, from Skalar Analytical), sodium hydroxide (Chemsolute, Th. Geyer GmbH, Renningen, Germany), hydrochloric acid (J.T. Baker, via Fisher Scientific), chloramine-T trihydrate (Merck), 1,3-dimethyl barbituric acid (Sigma-Aldrich, via Merck), and 4-pyridine carboxylic acid (Merck). Ultrapure water with a resistivity of > 18.2 MΩ cm was prepared by deionization of tap water using a Purelab Ultra device (ELGA Veolia, Celle, Germany).

Certified cyanide standard solutions were obtained from three different providers (certified concentrations in brackets, prepared from potassium cyanide salt of > 96% purity in 0.1% sodium hydroxide solution in purified water): Alfa Aesar, via Fisher Scientific (1000 ± 5 mg L^−1^); ULTRA Scientific, via Agilent, Waldbronn, Germany (1005 ± 6 mg L^−1^); and LGC Standards GmbH, Wesel, Germany (1000 mg L^−1^ ± 0.5% relative uncertainty). Each calibration prepared with a certified cyanide standard from one source was verified by analysis of diluted quality control standards derived from a certified standard from a second source.

Reagent solutions were prepared following the instructions of the CFA instrument SAN++ method for the analysis of free distillable cyanide (Skalar Analytical 2015). Compositions are based on ISO 14403-2 and are given there in detail (ISO 2012). However, as described above, polyoxyethylene dodecylether was substituted by the same volume of FFD6 solution for preparation of the buffer solution.

The prepared solutions were stored at room temperature in the dark and refreshed weekly according to the instructions in ISO 14403-2 (ISO 2012).

Gas bubbles in the solutions passing the glass fiber detection cell of the CFA instrument may reduce sensitivity by increasing background noise and thus the LOQ. To avoid this effect, all solutions were percolated with helium before a measurement series in addition to a treatment by sonication as recommended by the manufacturer.

### Analytical method

**Fig. 1:**
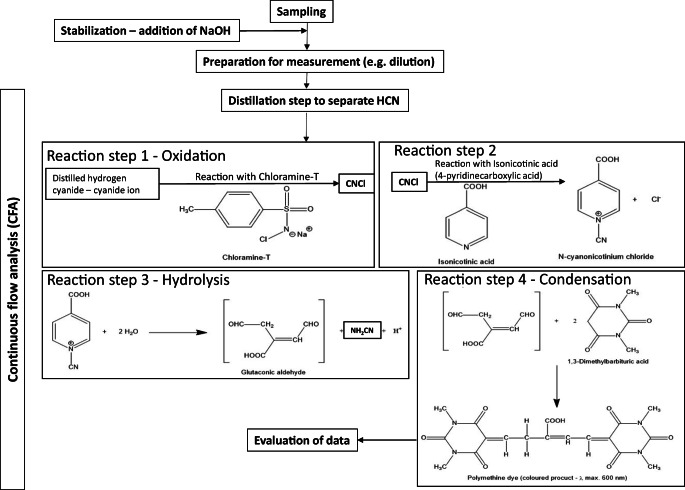
Schematic diagram of the analytical procedure (from sampling to evaluation of data) and the chemical reactions for the dye formation (Maranna et al. 2018; modified scheme)

The CFA method for the determination of free cyanide is described in detail in standard ISO 14403-2 (ISO 2012). In brief, first, zinc sulfate solution is added to the water sample for precipitation of iron cyanides as zinc cyanoferrate complexes. Then, the solution is heated to 125 °C at a pH of 3.8 to separate and enrich the free cyanide as hydrogen cyanide by distillation. The distilled hydrogen cyanide reacts with chloramine-T yielding cyanogen chloride which then forms a purple dye with pyridine-4-carboxylic acid (isonicotinic acid) and 1,3-dimethyl barbituric acid. The formed dye allows the sensitive photometric detection at 600 nm with an appropriate instrument (see Fig. 1).

For the basic validation (calculation of lowest possible LOD and LOQ), the CFA instrument was calibrated with the following equidistant cyanide standard concentrations (dilutions of a certified cyanide standard; dilution factor of high to low standard not more than 1:100): 0.000 (0.01 M sodium hydroxide in ultrapure water), 0.100, 0.200, 0.300, 0.400, 0.500, 0.600, 0.700, 0.800, 0.900, and 1.000 μg L^−1^. All standards were filled up with a 0.01 M sodium hydroxide solution (in ultrapure water). For all further measurement series (reproducibility, precision, environmental samples, etc.), calibrations in the relevant concentration ranges were performed (e.g., with calibration points of 0.000, 0.100, 0.250, 0.500, 0.750, 1.000, 1.250, and 1.500 μg L^−1^). The accuracy of the applied pipettes was verified regularly. The diluted standards were prepared freshly on a daily basis.

The CFA instrument’s software was used for the calculation of the calibration lines from the measured diluted standards (linear regression; correlation coefficients at least 0.995). Cyanide concentrations of investigated water samples were calculated by the software from the daily measured calibration line.

### Sample stabilization and storage

Aqueous solutions (including calibration standards) were stabilized by alkalinization to prevent losses of free cyanide. Following the procedure of the ISO 14403-2 standard (ISO 2012), samples were stabilized immediately after sampling by adjusting the pH to > 12 (addition of small volumes of 0.01 M sodium hydroxide solution). According to the ISO 14403-2 standard, the pH-adjusted samples are stable for up to 1 week. Furthermore, the stability for up to 1 week could be verified in stabilized free cyanide-spiked samples in the present study.

River and barrier lake water samples were taken at different seasons in 2016. They were stabilized as described above and stored at 4 °C in the dark. The appropriateness of these conditions was tested and confirmed by analysis of positive controls containing low amounts of free cyanide stored in the same manner.

### Prevention of interferences

Oxidizing compounds, or sulfides at concentration > 100 mg L^−1^, might interfere with the formation of the dye needed for photometric determination of free cyanide according to ISO 14403-2 (ISO 2012). Therefore, possible oxidizers were determined in samples as described in ISO 14403-2 (ISO 2012) by using potassium iodide starch paper (Macherey-Nagel, Düren, Germany). No staining of the paper was observable in any sample. Total sulfur concentrations of only 100–700 μg L^−1^ were measured in samples by ICP-OES following standard ISO 11885 (ISO 2007). Thus, no interferences were expected from oxidizers or from the detected sulfur compounds in the samples.

### Validation of the analytical method

For method validation, the following parameters were determined: limits of detection and quantification (LOD, LOQ), selectivity, reproducibility, repeatability, measurement uncertainty, and performance in an interlaboratory comparison. The validation followed the requirements of standard ISO/IEC 17025 (ISO/IEC 2005). Moreover, the method was tested during sampling campaigns with field samples.

## Results and discussion

### Validation

#### LOD and LOQ determination

A main objective in method validation is the determination of the LOD and LOQ. According to general WFD quality requirements, an LOQ of < 30% of the EQS has to be achieved (EC 2009). Since the most recent proposal for a potential EQS for free cyanide (Loos et al. 2018) considered a safety factor of 2 to the PNEC value of 1 μg L^−1^, the targeted LOQ level of the method was a magnitude below the PNEC (i.e., about 0.15 μg L^−1^).

Determinations of the LOD and LOQ (calculated as 3 * LOD) were performed according to the German standard DIN 32645 (DIN 2008). The respective principles that are described in Geiss and Einax (2001) allow the LOD determination via two alternative approaches. The direct method for LOD determination is based on the analysis of blanks. The indirect method is based on the data of a calibration line and thus yields an LOD for the selected working range including the uncertainty of the calibration.

The applied method blanks for the direct LOD determination method consisted of ultrapure water which was alkalinized with sodium hydroxide solution to a target pH value of 12. Two separate basic LOD determinations according to DIN 32645 (DIN 2008) were performed, so that two values were obtained. Measurements under optimal laboratory conditions led to LODs of 0.016 and 0.028 μg L^−1^ cyanide, and LOQs of 0.053 and 0.092 μg L^−1^ cyanide (95% confidence level).

For the indirect method for the determination of the LOD, the calibration data were evaluated. The calibration range was lower than the working range for measurements to be performed later in real environmental samples. Therefore, the indirect method provides also a lower LOD compared to the LOD gained for higher calibration ranges. In the indirect method, LODs/LOQs calculated according to DIN 32645 (DIN 2008) were 0.025/0.037 μg L^−1^ (two individual measurement series) and 0.050/0.074 μg L^−1^, respectively. These calculations were performed for a confidence level of 95%. For both methods (direct and indirect), comparable LODs and LOQs were obtained as required in DIN 32645 (DIN 2008), proving that a required LOQ of at least 0.15 μg L^−1^ for the quantification of free cyanide is achievable with the CFA system San++. Optimal conditions were applied for the measurements used for calculation of LOD and LOQ (e.g., degassing with helium, use of FDP6 as tenside, blank sample inserted after each sample).

For all further measurements for method validation, the LODs and LOQs were calculated by the indirect method (calibration method) for each measurement series. According to these results, a reproducible LOQ of 0.1–0.3 μg L^−1^ for the quantification of free cyanide using this CFA method can be obtained under realistic routine laboratory conditions.

#### Selectivity

The selectivity for photometric detection of free cyanide at the specific wavelength of 600 nm is based on a reaction of cyanide yielding a specific dye according to international standard DIN EN ISO 14403-2.

#### Precision and repeatability

For investigation of the precision, diluted alkalinized aqueous certified standards were measured. The limits of recoveries were set to 100 ± 15% for values equal to or above 2 times the concentration of the LOQ level and 100 ± 30% for concentration values above LOD but below concentrations of 2 times the LOQ level. To check the variance precision, samples with nominal concentrations of 0.100 μg L^−1^ (required recovery limits 100 ± 30%) and 0.75 μg L^−1^ (100 ± 15%) were measured (with *n* = 10 each). The precision results for a confidence level of 95% were 0.126 ± 0.007 μg L^−1^ (nominal 0.100 μg L^−1^ cyanide) and 0.724 ± 0.025 μg L^−1^ (nominal 0.750 μg L^−1^ cyanide).

To determine the repeatability, samples with nominal concentrations of 0.100 μg L^−1^, 0.250 μg L^−1^, 0.500 μg L^−1^, and 0.750 μg L^−1^ cyanide were investigated (*n* = 20, respectively). Mean measured values were 0.107 ± 0.019 μg L^−1^, 0.187 ± 0.019 μg L^−1^, 0.548 ± 0.029 μg L^−1^, and 0.724 ± 0.025 μg L^−1^. The nominal 0.100 μg L^−1^ cyanide concentration level was below the LOQ concentration but above the LOD concentration so that the quality control parameters were set to 100 ± 30%, whereas at other concentrations, recoveries of 100 ± 15% were required.

As the recoveries of all measurements were within the required range for the defined parameters, it was concluded that the method allows to quantify cyanide with the required precision and repeatability.

#### Reproducibility

Three measurement series were performed by three different operators on different days for determining the reproducibility of the method. The quality criteria for the relative standard deviation (RSD) for levels above the LOQ were set as maximum of 10% for a sufficient reproducibility (20% for levels < LOQ but > LOD). Diluted certified cyanide standards containing 0.100 μg L^−1^ (< LOQ but > LOD) and 0.500 μg L^−1^ (> LOQ) were measured with *n* = 10 each. For the individual measurements, RSD was in the range of 7.09–18.4% for 0.100 μg L^−1^ and 2.18–5.15% for 0.500 μg L^−1^ cyanide, respectively. The total RSD over all samples (*n* = 30 in total) was 19.1% (0.100 μg L^−1^) and 6.34% (0.500 μg L^−1^). The requirements for reproducibility were fulfilled. As the reproducibility is within the range of the quality assurance requirements for the defined parameters, it is concluded that the method allows to quantify cyanide with the required precision and reproducibility.

The laboratory participated in an interlaboratory comparison organized by IFA (Department of Agrobiotechnology, Tulln, Austria). Two samples of cyanide-spiked synthetic water-simulating environmental conditions were received. The nominal values for cyanide of these samples were 0.056 ± 0.002 mg/L and 0.042 ± 0.002 mg/L. After analyzing with the final protocol, 0.054 ± 0.006 mg/L and 0.043 ± 0.004 mg/L cyanide were found with the CFA method (recoveries of 96% and 102%). The interlaboratory comparison yielded excellent *z*-scores of − 0.20 and 0.13, respectively, for the samples (*z*-scores > − 2 < z < 2 are regarded as satisfactory).

#### Measurement uncertainty

The measurement uncertainty was determined according to the NORDTEST procedure (Magnusson et al. 2012). The NORDTEST method considers both the bias of the measurement (random effects) and the bias of the laboratory (possible systematic error) for the calculation of measurement uncertainty. Extended measurement uncertainties (coverage factor of 2) of ± 41% for samples between 2 times the concentration of the LOD and the LOQ and of ± 21% for samples with concentrations above the LOQ were determined for the applied CFA method.

#### External review

After completion of the method validation, DAkkS (Deutsche Akkreditierungsstelle, i.e., Germany’s National Accreditation Body) inspected the laboratory and the regarding written documents to examine a possible accreditation of the CFA method for determination of free cyanide. After review, the laboratory received the accreditation according to ISO/IEC 17025 (ISO/IEC 2005) for the implemented method for analysis of free cyanide.

The summarized validation information is shown in Table S1 (Supplementary Information).

### 1st field test

For testing the validated method in natural surface waters, samples from two sampling campaigns performed within 1 month during the winter/spring season covering five different sampling sites from the river Lenne, Germany were applied. Sampling sites were at the spring (#1), upstream the industrial area of the city of Schmallenberg (#2), the industrial area itself (#3), downstream the industrial area (#4), and downstream sampling site no. 4 (#5). Geo-coordinates are given in Table S2 (Supplementary Information).

Table 1 shows the amount of free cyanide in samples stabilized at a pH value of 12, measured 24 h after sampling (storage at 4 °C in the dark) of the 1st field test. The concentrations of free cyanide in the natural water samples from river Lenne were low and mainly below the calculated LOD/LOQ of the respective measurement series. Therefore, values below LODs were set to the respective LOD, while for values between LOD and LOQ, the actual measured concentrations are reported. The results prove that a realistic LOQ in the range of 0.1–0.3 μg L^−1^ during daily routine analysis of environmental samples from freshwaters can be achieved with the implemented CFA method.

**Table 1:** Free cyanide concentrations in samples from river Lenne at different sampling sites during the two sampling campaigns of the 1st field test (measured 24 h after sampling, stabilized at pH 12 and stored at 4 °C in the dark)

Sampling site no.	LOD (μg L^−1^)	LOQ (μg L^−1^)	Measured free cyanide concentration (μg L^−1^)		Reported free cyanide concentration (μg L^−1^)^#^	Range of extended measurement uncertainty (±)
1st sampling campaign (February/March sampling)						
1	0.051	0.188	0.034	< LOD	0.051	$
2	0.033	0.124	0.115	< LOQ	0.115	0.047
3	0.060	0.232	0.129	< LOQ	0.129	0.053
4	0.060	0.232	0.101	< LOQ	0.101	0.041
5	0.033	0.124	0.135	0.135	0.135	0.028
2nd sampling campaign (March sampling)						
1	0.112	0.461	0.067	< LOD	0.112	$
2	0.042	0.154	0.105	< LOQ	0.105	0.043
3	0.046	0.168	0.156	< LOQ	0.156	0.064
4	0.046	0.168	0.158	< LOQ	0.158	0.065
5	0.042	0.154	0.085	< LOQ	0.085	0.035

^#^Concentrations < LOD were set to the respective LOD of the measurement series; for concentrations between LOD and LOQ, the measured concentrations are given

^$^For concentrations below LOD, no measurement uncertainty could be calculated

### 2nd field test

After the 1st field test, a second approach was performed with samples from a barrier lake (Esmecke Stausee, two sampling sites) as well as from two sampling sites of the 1st field test during the fall season. Sampling sites were at Esmecke Barrier Lake (sites A and B), and at the river Lenne downstream the industrial area of Schmallenberg (site C; similar to site #4 of the 1st field test) as well as further downstream sampling site C (D; similar to site #5 of the 1st field test). Geo-coordinates are given in Table S3 (Supplementary Information). As for the 1st field test, measurements were divided into two campaigns. For the 1st sampling campaign of the 2nd field test, samples were taken at different times of the day to investigate daytime effects, whereas for the 2nd campaign, only samples taken around midday were considered.

Table 2 shows the amount of free cyanide in samples stabilized at pH 12, measured 24 h after sampling (storage at 4 °C in the dark) of the 2nd field test. The concentrations of free cyanide in the natural water samples from the barrier lake and the river Lenne were low and in some cases below the calculated LOD/LOQ of the respective measurement series. Therefore, values below LODs were set to the respective LOD, while for values between LOD and LOQ, the actual measured concentrations are reported. The results of the 2nd field test confirm the estimation of the first field test that a realistic LOQ in the range of 0.1–0.3 μg L^−1^ during daily routine analysis of environmental samples from freshwaters can be achieved with the implemented CFA method. For the daytime variations of the measured free cyanide concentrations seen in the 2nd sampling campaign, no clear trend was observed and the concentration differences between the samplings at each site did not exceed the respective ranges of the measurement uncertainties.

**Table 2:** Free cyanide concentrations in samples from Esmecke Barrier Lake and river Lenne at different sampling sites during the two sampling campaigns of the 2nd field test (measured 24 h after sampling, stabilized at pH 12, and stored at 4 °C in the dark)

Sampling site	Time of day	LOD (μg L^−1^)	LOQ (μg L^−1^)	Measured free cyanide concentration (μg L^−1^)		Reported free cyanide concentration (μg L^−1^)^#^	Range of extended measurement uncertainty (±)
1st sampling campaign (October sampling, different day times)							
A	Morning	0.052	0.192	0.244	0.244	0.244	0.051
	Midday	0.052	0.192	0.269	0.269	0.269	0.056
	Evening	0.052	0.192	0.185	< LOQ	0.185	0.080
B	Morning	0.029	0107	0.260	0.260	0.260	0.054
	Midday	0.029	0107	0.228	0.228	0.228	0.047
	Evening	0.029	0107	0.231	0.231	0.231	0.048
C	Morning	0.022	0.081	0.161	0.161	0.161	0.034
	Midday	0.022	0.081	0.192	0.192	0.192	0.040
	Evening	0.022	0.081	0.157	0.157	0.157	0.033
D	Morning	0.067	0.247	< LOQ	0.123	0.123	0.054
	Midday	0.067	0.247	< LOQ	0.142	0.142	0.062
	Evening	0.067	0.247	< LOQ	0.117	0.117	0.051
2nd sampling campaign (November sampling, only at midday)							
A	Midday	0.030	0.110	0.240	0.240	0.240	0.050
B	Midday	0.030	0.110	0.204	0.204	0.204	0.042
C	Midday	0.030	0.110	0.156	0.156	0.156	0.033
D	Midday	0.030	0.110	0.174	0.174	0.174	0.036

^#^Concentrations < LOD were set to the respective LOD of the measurement series; for concentrations between LOD and LOQ, the measured concentrations are given

^$^For concentrations below LOD, no measurement uncertainty could be calculated

## Conclusions and outlook

Quantification of low free cyanide in samples from freshwater systems was successfully performed applying the slightly modified CFA method on an instrument equipped with a 50-cm glass fiber cuvette. The achieved LOQs during the basic validation were in the range of 0.048–0.132 μg L^−1^ free cyanide.

According to the measurement of free cyanide in environmental samples, a reproducible LOQ of 0.1–0.3 μg L^−1^ can be obtained under realistic laboratory conditions. One LOQ of 0.461 μg L^−1^ shown in Table 1 is assumed to be an outlier.

The measurements prove that an LOQ for a potential compliance monitoring of free cyanide according to WFD requirements in concentrations of one order of magnitude below the current PNEC for freshwater organisms (1 μg L^−1^; ECHA (2020)) is achievable with the optimized CFA method presented here and the instrument with the 50-cm cuvette.

To test the implemented CFA method with real environmental samples, water from two field tests at a barrier lake and the river Lenne were taken for free cyanide analysis. Industry, which is working with cyanide, is not known to be located at the Esmecke Barrier Lake and the investigated stretch of the river Lenne. Both are in a rural area and expected to be uncontaminated. Concentrations in samples of the 1st field test during the winter/spring season were mostly below LOD or LOQ (Table 1) in contrast to most of the samples of the 2nd field test during the fall (Table 2). However, for concentrations between LOD and LOQ, the presence of free cyanide is confirmed in the sample but cannot be quantified reliably. Therefore, these values are given as the measured concentration ± measurement uncertainty. The low free cyanide concentrations found were probably originating from environmental processes generating cyanide (Jaszczak et al. 2017) or atmospheric input (ECETOC 2007).

Implemented and validated here, and under field conditions tested, CFA method allows to quantify low amounts of free cyanide in freshwater systems for environmental monitoring at sub-PNEC concentrations with reliable measurements down to levels of about 0.1 μg L^−1^. Thus, the method can be applied for a possible WFD watch list monitoring of free cyanide as suggested by Loos et al. (2018). However, in order to allow an assessment of the generated monitoring data, it is necessary to reliably determine relevant natural impact factors on free cyanide background concentrations in different water bodies, like season, climate, geology, light exposure, and water depths or other water body characteristics. Such data on environmental free cyanide levels will form the inevitable basis for a valid differentiation of anthropogenic pollution from natural fluctuations.

## Supplementary information

ESM 1 (DOCX 43 kb)

## Data Availability

The datasets supporting the conclusions of this article are included within the article and its additional file.
