# Supplementary material for: Determination of low environmental free cyanide concentrations in freshwaters
Source: Environ Sci Pollut Res Int. 2020 Dec 17;28(13):16244–52. doi: 10.1007/s11356-020-12062-7 (PMC7969539; doi:10.1007/s11356-020-12062-7)
Supplement: Supplementary file 1 — (DOCX 43 kb) [file 11356_2020_12062_MOESM1_ESM.docx]

**Supplementary Information for:**

**Determination of low environmental free cyanide concentrations in freshwaters**

Burkhard Knopf^*1^, Heinz Rüdel^1^, Dirk Hansknecht^1^, Thorsten Klawonn^1^, Knut Kreuzer^2^

1 Fraunhofer Institute for Molecular Biology and Applied Ecology IME, Schmallenberg, Germany
2 Röhm GmbH, Deutsche-Telekom-Allee, Darmstadt, Germany

Corresponding author:
Burkhard Knopf, e-mail: [burkhard.knopf@ime.fraunhofer.de](mailto:burkhard.knopf@ime.fraunhofer.de); phone +49 2972 302 208; fax +49 2972 302 319

**Table S1** Compilation of validation parameters and results (confidence level 95 %). Concentrations given are for free cyanide as determined with the CFA method; RSD - relative standard deviation

| **Validation parameter** | **Required value / range** | **Actually yielded value / range** |
| --- | --- | --- |
| **LOD / LOQ**  (two independent measurements and calibrations were performed) | LOD: < 0.15 µg L^-1^  (< 30 % of a potential EQS level)^§^ | LOD (direct method^$^): 0.02/0.03 µg L^-1^  LOQ (direct method): 0.05/0.09 µg L^-1^  LOD (indirect method^$^): 0.03/0.04 µg L^-1^  LOQ (indirect method): 0.09/0.13 µg L^-1^  **A realistic LOQ in routine analysis is estimated to be 0.1 - 0.3 µg L^-1^** |
| **Selectivity** | Distinct detection of analyte | **Specific formation of a dye for photometric quantification** |
| **Precision** | 100 ± 15 % for concentrations > 2 LOQ  100 ± 30 % for concentrations < 2 fold LOQ or < LOD |  |
|  | **Variance precision**  0.750 µg L^-1^: 0.64 – 0.86 µg L^-1^  0.100 µg L^-1^: 0.07 – 0.13 µg L^-1^ | Nominal 0.750 µg L^-1^ (n = 10):  0.724 ± 0.025 µg L^-1^  Nominal 0.100 µg L^-1^ (n = 10):  0.126 ± 0.007 µg L^-1^ |
|  | **Repeatability:**  0.100 µg/L: 0.07 – 0.13 µg L^-1^  0.250 µg/L: 0.21 – 0.29 µg L^-1^  0.500 µg/L: 0.43 – 0.58 µg L^-1^  0.750 µg/L: 0.64 – 0.86 µg L^-1^ | Nominal 0.100 µg L^-1^ (n = 20):  0.107 ± 0.019 µg L^-1^  Nominal 0.250 µg L^-1^ (n = 20):  0.187 ± 0.019 µg L^-1^  Nominal 0.500 µg L^-1^ (n = 20):  0.548 ± 0.029 µg L^-1^  Nominal 0.750 µg L^-1^ (n = 20):  0.724 ± 0.025 µg L^-1^ |
| **Reproducibility** | RSD <20% for values < LOQ  RSD <10% for values > LOQ | Nominal 0.1 µg L^-1^ (n = 10): 1^st^ measurement: 7.09 % 2^nd^ measurement: 18.4 % 3^rd^ measurement: 12.4 % Total (n = 30): 19.1 %  Nominal 0.5 µg L^-1^ (n =10): 1^st^ measurement: 2.18 % 2^nd^ measurement: 5.15 % 3^rd^ measurement: 3.95 % Total (n = 30): 6.34 % |
|  | **Participation in interlaboratory comparison**  z-score < 2 is satisfying  z-score of < 2 z < 3 is questionable  z score < 3 is not satisfying | Two different samples  Z scores of -0.20 and 0.13 (satisfying) |
| **Measurement uncertainty^#^** | **< 50 % of a potential EQS level^§^** | Extended measurement uncertainty  (k = 2): < LOQ and > LOD: 41%  > LOQ: 21% |

^$^ Determination of LOD/LOQ according to DIN 32645 (DIN 2008);  ^#^ Determined according to the NORDTEST approach (Magnusson et al. 2012);
^§^ According to EC (2009) and Loos et al. (2018).

**Table S2** Description and geo-coordinates (longitude/latitude) of sampling sites at river Lenne, North-Rhine Westphalia, Germany

| Sampling site no. | Description | Latitude | Longitude |
| --- | --- | --- | --- |
| 1 | “Kahler Asten”  Spring | 51.179323 | 8.484472 |
| 2 | Gleidorf  Upstream the industrial area | 51.161973 | 8.315074 |
| 3 | Industrial area  Downstream the industrial area | 51.154175 | 8.295024 |
| 4 | Spa gardens  Downstream the industrial area | 51.147756 | 8.288797 |
| 5 | Mill  Downstream sampling site no. 4 | 51.142228 | 8.284759 |

**Table S3** Description and geo-coordinates (longitude/latitude) of sampling sites of the 2^nd^ field test at Esmecke Barrier Lake and river Lenne, North-Rhine Westphalia, Germany

| Sampling site | Description | Latitude | Longitude | Respective site no. 1^st^ field test |
| --- | --- | --- | --- | --- |
| A | Esmecke Barrier Lake;  first sampling area | 51.29575 | 8.158666 | - |
| B | Esmecke Barrier Lake; second sampling area | 51.2951111 | 8.157138 | - |
| C | Spa gardens, downstream the industrial area | 51.147756 | 8.288797 | 4 |
| D | Mill, downstream sampling site C | 51.142228 | 8.284759 | 5 |
